# Night running and night cycling: a review of sociological drivers, health benefits, and their interaction with urban green spaces

**DOI:** 10.3389/fpubh.2025.1559048

**Published:** 2025-03-20

**Authors:** Jiayi Zhang, Xiaoyu Yang, Tianhang Peng

**Affiliations:** ^1^School of Sports Science, Beijing Sport University, Beijing, China; ^2^University of Science and Technology Beijing, Beijing, China; ^3^Henan University, Kaifeng, China

**Keywords:** night running, night cycling, public health, urban green spaces, city infrastructure

## Abstract

This study investigates the rising trends, sociological drivers, health benefits, and the interaction of night running and night cycling with urban green spaces. Findings indicate that night running and night cycling are mainly driven by increased health awareness, changes in work patterns, social media promotion, and urban economic development. Physiologically, night running and cycling enhance cardiovascular function, alleviate stress, and improve sleep. However, air pollution, inadequate infrastructure, and safety concerns limit the sustainability of these activities. Urban green spaces play a crucial role in promoting nocturnal exercise, with improvements in lighting, safety, and recreational areas due to the growing demand for night fitness. Governments are encouraged to enhance infrastructure and optimize green space planning to foster healthier urban environments.

## Introduction

In recent years, the popularity of nocturnal exercise has surged due to various socio-cultural and economic factors. Specifically, night running and night cycling have become significant components of urban fitness routines (1). These activities, which involve moderate to high-intensity exercise performed during the night, have become essential for those looking to balance the demands of work, social lives, and health. Studies indicate that exercising in urban green spaces, especially at night, can offer substantial physical and mental health benefits, ranging from improved cardiovascular health to stress relief and enhanced sleep quality (2, 3). However, despite these advantages, the growth of night fitness is not without its challenges, including safety concerns, poor infrastructure, and air pollution. The interaction between urban green spaces and nocturnal exercise is also an important area of focus, as improvements in public parks and recreational areas have been shown to directly influence participation in such activities. This review aims to examine the sociocultural drivers of night running and cycling, their health benefits, the relationship between nocturnal fitness and urban green spaces, and the role of governmental policies and infrastructure in supporting these activities. It uses literature review, data analysis, and case studies to explore these aspects and provides policy recommendations for urban planning and public health.

## Sociological drivers

Night running and cycling are increasingly linked to the growing awareness of health, shifts in lifestyle, and social demands. These activities require minimal equipment, are adaptable, and offer flexibility, catering to urban populations seeking both health benefits and social interaction (4). The rise of social media and health education has heightened awareness of the benefits of regular exercise, particularly those that promote social interaction (5).

Work pressure and flexible work systems have led many urban residents, particularly those in high-stress jobs, to turn to nocturnal exercise for stress relief (4). The quieter night roads provide a safer environment, with surveys showing that over 60% of Asian city residents exercise at night due to time constraints, with night running and cycling as the preferred activities (6, 7). Night running offers more flexibility and social interaction compared to morning runs, although it presents unique challenges (8, 9) (Supplementary Table 1).

Psychological regulation is a key motivator for nocturnal exercise. Night running and cycling significantly reduce anxiety in high-stress workers (10). According to Self-Determination Theory, individuals are inclined to choose exercise forms that offer high autonomy and social interaction. Night running and night cycling not only provide flexibility in adjusting the pace of exercise but also enhance social support and group cohesion through community activities. The Health Belief Model suggests that an individual’s health behavior is influenced by perceived health threats, perceived benefits, barriers, and self-efficacy. Awareness that night running and night cycling can alleviate stress increases participation motivation and strengthens self-efficacy (11). A European study showed that night running communities experienced a 30% reduction in anxiety and a 50% increase in exercise adherence (10).

Social media has accelerated the growth of nocturnal fitness communities (12). Increased engagement with social platforms correlates with higher participation in nocturnal exercise. Online clubs and forums encourage offline group activities, and platforms like Strava and Nike Run Club provide data tracking, social interaction, and motivation. During COVID-19, global participation in night running increased by 65%, with over 60% of new participants influenced by online communities (5).

## Physiological benefits

Night running and cycling provide significant physiological benefits, including increased metabolic rate, cardiovascular adaptation, and regulation of the nervous system to improve mood and sleep quality (Supplementary Table 2). These activities increase daily energy expenditure and promote fat oxidation. When combined with reduced caloric intake, they significantly improve body fat percentage (13). Nocturnal exercise increases energy expenditure by 12–15%, with night running yielding particularly prominent results (14).

Night running and cycling reduce blood pressure, enhance vascular elasticity, and improve heart rate variability (HRV) (15). Night running helps reduce sympathetic nervous system activity, promotes parasympathetic regulation, and improves cardiovascular resilience (7). Studies show that night runners experience an 18% increase in HRV and a 5–8 mmHg reduction in blood pressure (16). Cycling in cooler environments stabilizes heart rate and reduces cardiovascular strain caused by heat (17). However, to minimize air pollution risks, nocturnal exercise in areas with better air quality is recommended (5).

Moderate-intensity night running and cycling improve sleep quality, particularly when exercise is performed more than 90 min before sleep (18). These exercises improve sleep efficiency by 10–15% and increase deep sleep by 20 min, benefiting individuals with anxiety (5). The release of endorphins helps alleviate anxiety and depression, especially for high-stress groups (9). Night runners engaging in 3–4 weekly sessions see a 25% reduction in anxiety, a greater effect compared to morning exercisers (10), along with improved well-being (11). However, high-intensity exercise near bedtime can delay sleep onset and reduce sleep quality. It is recommended to maintain moderate intensity and avoid vigorous exercise within an hour of sleep (19, 20).

Night cycling, as a low-impact activity, is ideal for individuals with joint issues. Compared to running, cycling puts less strain on the knees, improves fat oxidation and energy expenditure, and helps optimize metabolic health (21). Cycling in cooler environments also boosts energy expenditure and muscle strength (15).

## Interaction with urban green space development

Night running and cycling exhibit a bidirectional interaction with urban green space planning. Enhanced accessibility and quality of nocturnal green spaces—defined as green areas such as parks and greenways that are optimized for nighttime exercise—directly elevate participation in these activities, while their popularity reciprocally drives infrastructure upgrades, establishing a health-environment-economy synergy (22). Green spaces are positively correlated with improved mental health, emotional regulation, and physical activity, particularly in high-stress professions, where they help reduce stress and improve mood (23). Residents living within 500 meters of green spaces are 20–30% more likely to engage in nocturnal fitness activities (2, 24). Cities with higher green space coverage show higher participation rates in night running and cycling and lower depression levels (25, 26). Green spaces also foster collective activities, strengthening community cohesion and correlating with lower crime rates in cities with active nocturnal exercise (27).

Limited visibility during nighttime exercise reduces sensory experiences, such as visual and olfactory stimuli, potentially diminishing health benefits (28). Nonetheless, nighttime running and cycling alleviate daytime traffic congestion and air pollution while driving improvements in greenway and sports infrastructure. For example, Chengdu’s development of a nighttime-friendly greenway network significantly increased residents’ nighttime activity levels and optimized urban green space use. The popularity of such activities raises the frequency of nighttime green space usage, fostering continuous upgrades in green infrastructure. Greenway investments yield a return of £2.5 for every £1 spent (29). Additionally, the rise in nighttime running and cycling enhances green space utilization, stimulates the urban nighttime economy, and drives green infrastructure’s economic operation, potentially increasing urban returns by 15–20% (30). Research from Latin American cities suggests that incorporating nighttime exercise into urban marketing strategies enhances green space commercial value and urban competitiveness (31). Furthermore, nighttime exercise contributes to the growth of the sports industry and related commercial facilities, such as fitness services and sporting goods.

Night running and cycling not only drive green space development but also improve urban environments through green commuting, promoting economic growth. Cities can capitalize on green space resources to create unique nocturnal fitness events, such as illuminated tracks or night marathons, attracting tourists and investors while supporting sustainable green space development.

## Constraints and development strategies of urban environment

Night running and cycling, crucial for urban healthy lifestyles, face constraints from safety issues, air pollution, infrastructure disparities, and social equity challenges.

Low visibility and sparse foot traffic increase the risks of traffic accidents and crime, with inadequate lighting being the main safety concern (32). Improving street lighting can enhance safety and reduce crime (33). Additionally, air pollution, especially from traffic, negatively affects the health benefits of nocturnal exercise. Air pollution is typically worse at night, especially during peak traffic hours, increasing health risks (34). Studies show that cities like Delhi have dangerously high concentrations of NOx and VOCs at night, posing respiratory risks to exercisers (35). Green buffers like green belts and plant walls help reduce the impact of air pollution (36). The uneven distribution of green spaces, particularly in low-income areas, limits opportunities for nocturnal exercise (37). Research in Chicago indicates that green space accessibility in low-income communities is 40–50% lower than in higher-income areas (38), with similar studies in Indonesia showing limited green space development in low-income regions restricting nocturnal fitness (39).

To promote the development of night running and cycling, strategies should include: enhancing night-time green space construction, installing smart lighting, planning exercise paths, and raising public awareness (40); using green buffers to reduce air pollution, monitoring air quality, and providing real-time data to optimize exercise timing (41); increasing green space investment in low-income areas to improve accessibility and promote public fitness (42); and using smart technologies like air pollution warning apps and fitness route planning systems to enhance the exercise experience. Community cooperation and volunteer patrols can also ensure safety during nocturnal exercise (43).

## Conclusion

**Figure 1:**
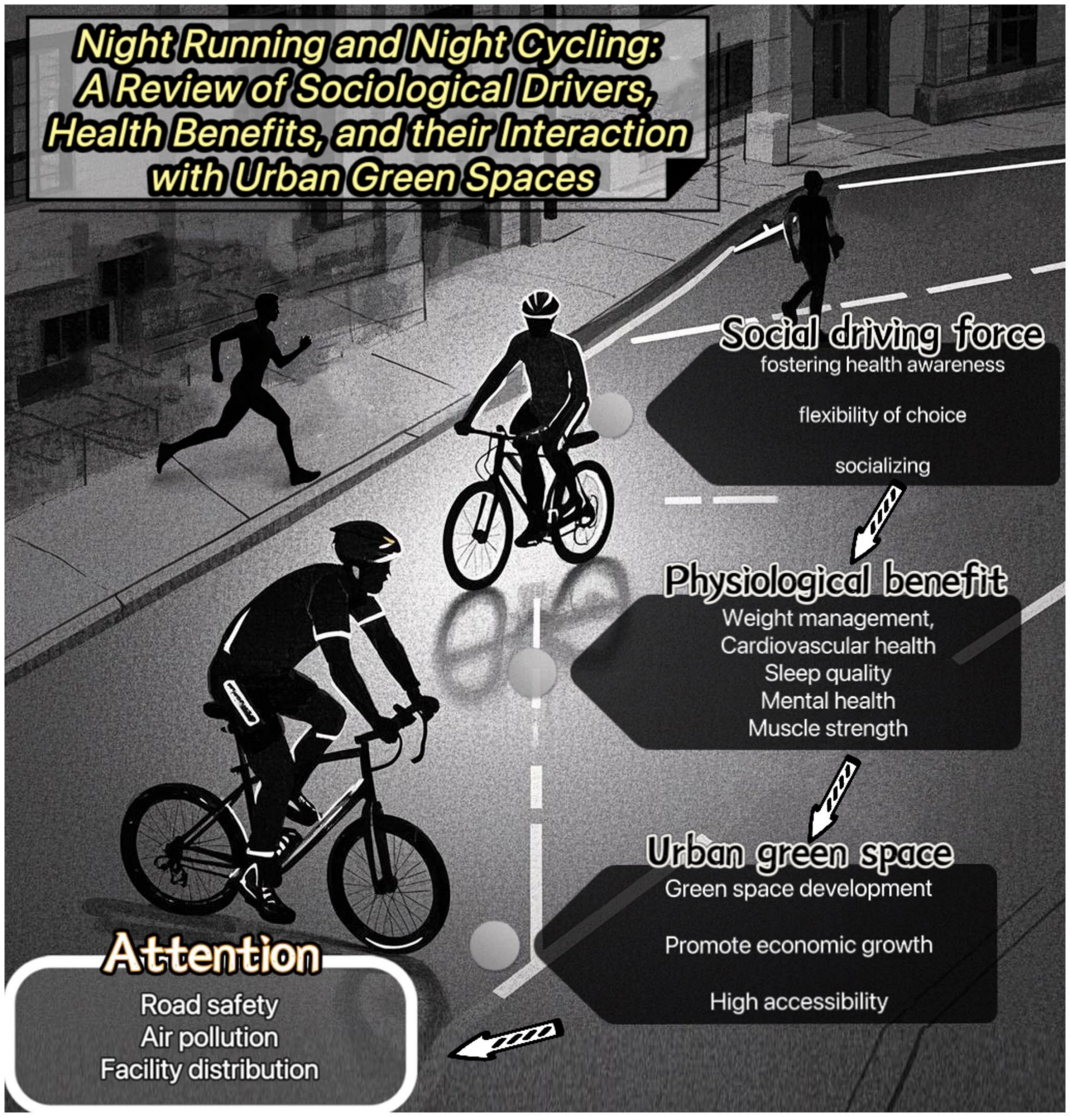
A relationship diagram of various factors.

The study underscores the significant role of night running and night cycling in promoting urban health, driven by growing health awareness, lifestyle changes, and urban economic growth (Figure 1). These activities offer considerable physical benefits, like cardiovascular improvement and better sleep quality. Urban green spaces play a pivotal role in enhancing the appeal and safety of nocturnal exercise, with accessibility and infrastructure improvements fostering increased participation. However, air pollution, safety concerns, and infrastructure disparities limit their potential. The integration of big data and smart technologies is recommended to optimize urban fitness paths and improve the nocturnal exercise experience. While based on literature and case studies, future research should involve field surveys and data analysis. Governments are encouraged to focus on optimizing green space planning and infrastructure to create healthier and more sustainable urban environments, benefiting both public health and economic vitality.
