# Supplementary material for: Night running and night cycling: a review of sociological drivers, health benefits, and their interaction with urban green spaces
Source: Front Public Health. 2025 Mar 20;13:1559048. doi: 10.3389/fpubh.2025.1559048 (PMC11965350; doi:10.3389/fpubh.2025.1559048)
Supplement: Supplementary file 1 [file Data_Sheet_1.docx]

**Supplementary Materials**

**Table 1. Comparison of Night Running and Morning Running**

| Dimension | Night Running | Morning Running |
| --- | --- | --- |
| **Main Motivation** | Stress relief, social interaction | Habit formation, boosting metabolism |
| **Target Population** | People with free time at night, busy workers | Early risers, those who exercise at fixed times |
| **Environmental Factors** | Reduced traffic, suitable temperature, but insufficient lighting | Good air quality, sufficient lighting, but low morning temperature |
| **Social Attributes** | More community activities, group running | Primarily individual training, less social interaction |

**Table 2. Physiological Health Impacts of Night Running and Night Cycling**

| Impact Category | Night Running | Night Cycling | Reference |
| --- | --- | --- | --- |
| **Energy Expenditure** | Immediately increased | Immediately increased | [Brito et al., 2022] |
| **Fat Oxidation** | High | Moderate | [Metabolism. 2003 Jun] |
| **Blood Pressure Change** | Decreased by 5-8 mmHg | Decreased by 4-7 mmHg | [Nuuttila et al., 2022] |
| **Heart Rate Variability (HRV)** | Increased by 18% | Increased by 15% | [Nuuttila et al., 2022] |
| **Sleep Efficiency** | Increased by 10%-15% | Increased by 8%-12% | [Aloulou et al., 2019] |
| **Anxiety Level, Negative Emotions** | Reduced | Reduced | [Davis et al., 2020] |
| **Joint Pressure** | High (greater impact) | Low (lesser impact) | [Li et al., 2023] |
| **Lower Limb Muscle Endurance Improvement** | Low | Increased by 15% | [Darch L et al.，2022] |
| **Hepatic Triglyceride Levels** | **Increased** | **Increased** | [Kovynev A et al.，2024] |

**Reference**：

1. Brito, L., Marin, T. C., Azevêdo, L., Rosa-Silva, J., Shea, S., & Thosar, S. (2022). Chronobiology of exercise: Evaluating the best time to exercise for greater cardiovascular and metabolic benefits. Comprehensive Physiology, 12(3), 3621-3639.
2. Achten J, Venables MC, Jeukendrup AE. Fat oxidation rates are higher during running compared with cycling over a wide range of intensities. Metabolism. 2003 Jun;52(6):747-52. doi: 10.1016/s0026-0495(03)00068-4. PMID: 12800102.
3. Nuuttila, O.-P., Seipäjärvi, S. M., Kyröläinen, H., & Nummela, A. (2022). Reliability and sensitivity of nocturnal heart rate and heart-rate variability in monitoring individual responses to training load. International Journal of Sports Physiology and Performance.
4. Aloulou, A., Duforez, F., Bieuzen, F., & Nédélec, M. (2019). The effect of night‐time exercise on sleep architecture among well‐trained male endurance runners. Journal of Sleep Research, 29.
5. Davis, M., MacCarron, A., & Cohen, J. (2020). Social reward and support effects on exercise experiences.
6. Darch L, Chalmers S, Causby R, Arnold J. Effect of Running-Induced Fatigue on Tibial Acceleration and the Role of Lower Limb Muscle Strength, Power, and Endurance. Med Sci Sports Exerc. 2023 Mar 1;55(3):581-589. doi: 10.1249/MSS.0000000000003062. Epub 2022 Oct 17. PMID: 36251400.
7. Kovynev A, Ying Z, Zhang S, Olgiati E, Lambooij JM, Visentin C, Guigas B, Ducarmon QR, Rensen PCN, Schönke M. Timing Matters: Late, but Not Early, Exercise Training Ameliorates MASLD in Part by Modulating the Gut-Liver Axis in Mice. J Pineal Res. 2024 Dec;76(8):e70003. doi: 10.1111/jpi.70003. PMID: 39539028.
